# Navigating Challenges in Mass Spectrometry Analysis of Endogenous and Synthetic Protein Modifications

**DOI:** 10.3390/biom16030367

**Published:** 2026-02-28

**Authors:** Caroline M. Hanson, Dina L. Bai, Jarrod A. Marto

**Affiliations:** 1 Department of Cancer Biology, Dana-Farber Cancer Institute, Harvard Medical School, Boston, MA 02215, USA; chanson@g.harvard.edu; 2 Department of Medicine, Harvard Medical School, Boston, MA 02115, USA; 3 Department of Chemistry, University of Virginia, Charlottesville, VA 22904, USA; dlb6z@virginia.edu; 4 Department of Biochemistry and Molecular Genetics, University of Virginia School of Medicine, Charlottesville, VA 22908, USA

**Keywords:** synthetic modification, database search, labeling profile, peptide-spectrum match (PSM), isobaric, collision induced dissociation (CID), electron capture dissociation (ECD), electron transfer dissociation (ETD), fragment remnant, diagnostic ion, immonium ion, precursor ion scanning, site-determining ion, positional isomer, chimeric spectra, dynamic exclusion, parallel reaction monitoring (PRM), error-tolerant search, open search

## Abstract

Mass spectrometry-based analysis of post-translational modifications (PTMs) is a key strategy for characterizing protein regulation and identifying disease-associated targets, with endogenous PTMs serving as biomarkers for disease diagnosis and therapeutic response. More recently, chemical proteomic strategies have adapted PTM-focused workflows to measure engagement of covalent and photoactivatable small-molecule probes, expanding the scope of ligand discovery for these disease-associated targets. This review provides an overview of mass spectrometry-based PTM analysis workflows, including LC–MS/MS acquisition and post-acquisition data processing, with an emphasis on how modification-specific physicochemical properties influence PTM detection and identification. Common analytical challenges that limit PTM identification, including variable MS/MS fragmentation behavior and modification site localization, are discussed using modifications such as phosphorylation and photoaffinity labeling probe adducts as representative examples. Recent advances in acquisition strategies and computational tools that improve spectral quality and confidence in PTM assignment are also summarized. Additionally, approaches for the analytical validation of modification events, such as metabolic labeling strategies, are described. Together, this review outlines key considerations, capabilities, and limitations of MS-based PTM profiling and provides a framework for interpreting PTM datasets to support their effective integration into downstream biochemical and disease target validation studies.

## 1. Introduction

Proteins are the physical actors mediating physiologic and pathologic cell processes. Protein function is often dictated by endogenous small molecules that transiently bind as ligands or covalently modify the protein as post-translational modifications (PTM). PTMs are a foundational mechanism of cellular regulation, dictating protein activity, stability, interactions, and localization. The enzymes that mediate PTM activity are often dysregulated in disease and therefore comprise attractive drug targets. Indeed, several new small molecules that inhibit kinases [1,2] or lysine acetyltransferases [3] are approved for clinical use or are undergoing clinical trials. As a result, understanding the landscape of PTMs can reveal how pathological insults commandeer endogenous cellular processes and provide biomarkers to monitor the therapeutic efficacy of treatment regimens.

**Figure 1:**
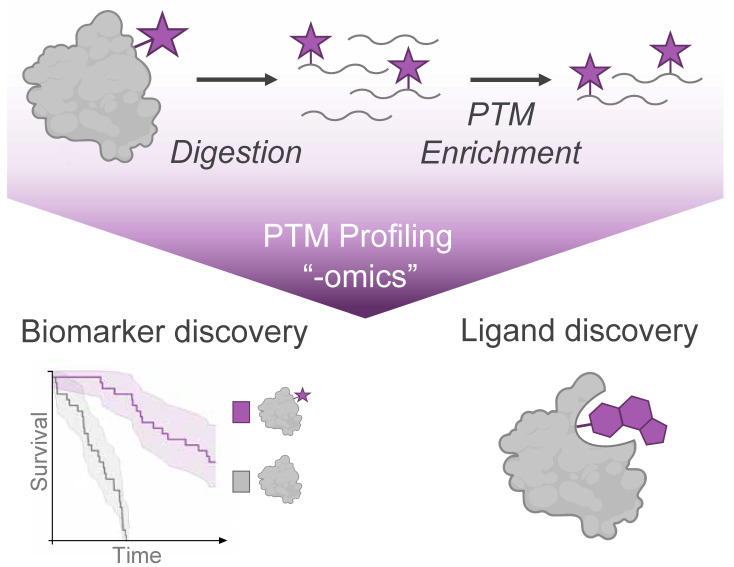
Mass Spectrometry (MS)-based profiling of protein modifications (purple star) enables the discovery of disease biomarkers from endogenous post-translational modifications and discovery of ligands from small molecule modifications of protein targets.

Mass spectrometry (MS) analysis has become the cornerstone of global PTM profiling, providing an unmatched combination of sensitivity and quantification for a wide array of protein modification events. MS-based proteomic analysis has characterized PTMs spanning diverse chemical classes, including phosphorylation, acylation, glycosylation, and ubiquitination, among many others. Over time, the development of PTM enrichment approaches has facilitated large-scale “-omics” profiling of these PTMs in cell lines, genetic models, and primary human tissue samples (Figure 1).

Phosphorylation is one of the most extensively profiled PTMs and serves as an exemplar of how PTM analysis can be translated into clinical applications. Phosphoproteome analysis has been instrumental in elucidating signal transduction pathways by mapping regulated phosphorylation sites in the context of cellular adaptation and disease. More recently, aberrant phosphorylation events have been explored as tractable footholds on therapeutic targets [4]. There are currently more than 80 FDA-approved protein kinase inhibitors [1], with many new kinase-targeted modalities entering clinical trials [2].

Dysregulated phosphorylation events, similar to other PTMs, also hold clinical utility as biomarkers for diagnosis, patient stratification, and as biochemical readouts of therapeutic efficacy in cancer, neurodegeneration, and other pathophysiologies. Advancements in phosphorylation analysis over the past three decades have enabled reproducible, in-depth profiling of the phosphoproteome, allowing functional interrogation of the cellular kinome to help predict patient response to these approved drugs [5]. For example, phosphorylation on AKT at Thr308 and Ser473 are used to monitor responses to kinase inhibitors in cell models of small cell lung cancer [6]. Similarly, MS-based assays quantifying tau phosphorylation in blood have been developed as a diagnostic tool for Alzheimer’s disease [7]. Together, these examples underscore the progress in translating PTM analysis technologies into pre-clinical and clinical settings [8]. Continued advances in methods and automation, MS instrumentation, and data analysis frameworks support statistically powered proteomic studies to inform patient stratification or prediction of therapeutic response.

The adoption of chemical proteomic approaches into the drug discovery process has further motivated developments in the field of MS-based PTM analysis. Here, mass spectrometry techniques are particularly powerful in the realm of covalent pharmacology, which relies on small molecules that covalently bind to and disrupt the activity of their target disease proteins. Screening campaigns may utilize covalent probes that irreversibly bind mechanism-related protein families, such as kinases. Chemical proteomics refers to mass spectrometry methods that leverage these probes to identify small molecule starting points for inhibitors and other drugs or characterize new pockets on proteins of interest. These covalently attached small molecules are protein modifications, similar to endogenous PTMs, that can be considered as “synthetic modifications”. Identification of probe-derived synthetic modification events enables identification of ligandable pockets on disease targets, accelerating drug design as well as our understanding of drug mechanism of action [9,10].

A common strategy in PTM profiling involves a “bottom-up” MS workflow, where proteins are first proteolytically digested into peptides for subsequent MS analysis. This approach is optimized for canonical or unmodified peptides; however, a key challenge in applying this technique to modified peptides lies in accounting for how the endogenous or synthetic modification adducts may alter a peptide’s physicochemical behavior during sample preparation and MSanalysis. Across a wide range of modifications, mass spectrometry researchers have improved PTM-peptide identifications through innovations in separation, acquisition, and data processing workflows that are often transferable to other modifications. Furthermore, PTM-profiling data and analyzed results have become publicly accessible, enabling the broader scientific community to generate hypotheses of novel therapeutic targets or biomarkers from these databases [11,12]. For these researchers, it can still be challenging to assess modification events of interest from these large MS datasets without an understanding of how the site was identified, along with the statistics that underlie confidence in the peptide sequence and PTM site assignments.

In this review, we highlight several key challenges of correctly identifying PTMs via MS in a manner accessible to researchers whose primary expertise lies outside protein MS. We describe approaches to (1) adapt conventional MS workflows to account for changes in physicochemical properties associated with these modifications and (2) validate specific modification events by MS. Optimization of MS acquisition and post-processing can improve the sensitivity of identification for endogenous PTMs in primary tissue samples, such as patient biopsies, that may have reduced protein content compared to samples prepared from cell line models. These optimized strategies also offer valuable insights for profiling emerging PTMs, such as synthetic modifications introduced via covalent chemical probes. For the broader scientific community, understanding the limitations and scope of MS-based PTM profiling approaches can inform interpretation of MS results and ultimately provide valuable data for disease target validation studies.

## 2. Overview & Limitations of PTM Analysis

PTM profiling experiments are typically performed using a bottom-up approach, where proteolytically digested peptides serve as the analytes for MS analysis. Both endogenous and synthetic protein modifications typically occur at low stoichiometry relative to their unmodified counterparts, making their detection in complex proteomes inherently challenging [13,14]. To address this limitation, modification-specific enrichment of PTMs from digested peptides is a critical component of sample preparation to provide sufficient material for acquisition of informative MS data (Figure 1). These enrichment strategies typically exploit distinct structural or physicochemical properties of the modification of interest using approaches such as immunoprecipitation, affinity chromatography, or, for synthetic modifications, covalent capture [14]. Ongoing efforts will continue to expand the repertoire of chemical and biochemical methods to enrich PTM-bearing peptides or proteins from complex samples, with comprehensive discussions of specific PTM enrichment strategies available in several excellent reviews [13,15,16].

**Figure 2:**
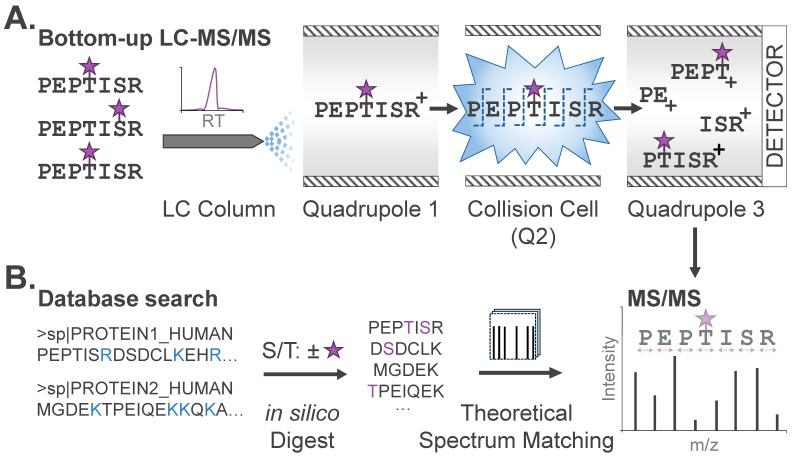
PTM profiling via bottom-up MS. (**A**) Modified peptides in complex mixtures are temporally separated by liquid chromatography (LC). PTMs are designated with purple stars. Peptides in a restricted mass-to-charge (*m*/*z*) region are isolated and subjected to fragmentation (MS/MS) and detection of fragment ions after amide bond cleavage across the peptide backbone. (**B**) Database search algorithms compare theoretical peptide sequences to observed MS/MS to identify candidate peptide sequences and their modifications. Protein sequences, digestion sites (blue), and potential modifications (purple) are typical input parameters specified by the user.

The enriched, modified peptides are then analyzed by liquid chromatography-tandem MS (LC-MS/MS). In brief, LC-MS/MS strategies provide temporal separation of peptide species by liquid chromatography coupled directly to a mass spectrometer. As peptides elute from the LC column and are ionized for introduction into the mass spectrometer, intact peptide mass-to-charge (*m*/*z*) ratios are first measured (‘MS’ or ‘MS1’ scan), followed by isolation and fragmentation of peptides for ‘MS/MS’, yielding unique fragment ion spectra for each peptide (Figure 2A). Peptide sequence can be determined through manual or automated interpretation of the precursor (MS1) peptide mass, along with the peptide fragment ions (MS/MS).

Peptide sequences and modification sites are commonly identified from LC-MS/MS data via database search, where each MS/MS spectrum is matched to theoretical spectra generated in silico from a user-specified database (e.g., human, mouse, etc.) of protein sequences (Figure 2B). Specific database search tools, including trade-offs and decision-making criteria for practitioners, have been previously described [17]. For expected modifications, users also specify the modification mass and labeling profile, or the scope of amino acid residues that could bear this modification. For phosphoproteome analysis noted above, users specify a net mass adduct of 79.9663 Da (PO_3_H) that could occur on serine, threonine, or tyrosine (S/T/Y). This input enables algorithms to generate a list of theoretical unmodified and modified peptides that comprise candidates for identification from the analyzed sample. Each experimental MS/MS spectrum is then matched to the most concordant peptide sequence by comparing the experimental precursor and fragment masses with those of theoretical candidate peptides. This is known as a peptide-spectrum match (PSM). For this reason, conventional database searches require specification of any expected PTMs; modifications not explicitly defined will not be identified, regardless of their abundance. For candidate peptides identified with modifications, a second stage of analysis utilizing PTM localization algorithms will then assign a relative confidence score for the modification existing at a given site. Typical outputs from a PTM profiling experiment will include the peptide sequence, modification, and site of modification, along with confidence scores corresponding to the peptide sequence and site of modification assignment. These datasets commonly encompass tens-of-thousands of unique peptide sequences along with modifications localized to specific amino acids.

It is important to note that database search algorithms are, while powerful, also inherently fallible. A central assumption of database searching is that the observed MS/MS spectrum corresponds to a peptide-modification combination present within the user-specified search space. When spectra arise from unanticipated (and therefore unspecified) modifications or protein sequences, these algorithms attempt to assign the closest available match, which can result in missed or incorrect identifications [18]. This risk is exacerbated in the case of low-abundance peptides, which typically yield fewer informative fragment ions to match. While search tools utilize conservative statistical thresholds to limit false positive identifications, a fraction of spectra in any database search output will remain misassigned. Consequently, manual inspection of selected MS/MS spectra is recommended for the subset of PTM assignments most relevant to the underlying biological question from a PTM profiling experiment.

As one of the most studied PTMs, phosphorylation is also a prime example of the unique challenges associated with identifying modified peptides. While modifications will increase peptide mass, knowledge of the mass shift alone is insufficient to provide unambiguous identification. First, different PTMs may be isobaric, or have nearly identical masses (e.g., phosphorylation vs. sulfonation) [19], while a single PTM located at different amino acids on the same peptide yields isomeric species that have identical masses. Either scenario can result in modification misassignment. Misassignments can also be caused by chemical artifacts, generated during sample preparation or during MS analysis, that mimic the mass shift of common PTMs [20].

Critically, modifications affect other peptide physicochemical properties, including retention on chromatographic separation systems as well as gas-phase fragmentation behavior during MS/MS. The magnitude of these effects is difficult to predict *a priori* but can confound sample preparation as well as acquisition and analysis of MS data. For example, phosphoryl groups modify proteins through labile phosphoester bonds that are prone to dissociate during MS/MS fragmentation, impeding correct identification and localization of phosphorylation events [21]. Furthermore, phosphorylation can occur on several amino acids, which makes it challenging to chromatographically resolve phospho-isoforms localized to adjacent residues or confidently assign a phosphorylation event to a specific residue during data analysis. These effects are exacerbated for synthetic modifications, where the high structural diversity of the covalent probes themselves and the scope of amino acids that these probes can modify limit standardized identification approaches. These challenges ultimately limit the ability of conventional MS acquisition and database searches to characterize modified peptides at the same depth or confidence level as their unmodified counterparts. For this reason, understanding modification-specific physicochemical properties informs interpretation of reported “-omics” datasets and facilitates validation of modification events of interest before further biochemical or clinical investigation. Rigorously characterizing the signature MS behavior of a modification type can be leveraged to improve identifications and build scalable platforms for quantitative profiling of novel modifications across the proteome [22].

## 3. Challenges in Fragmentation of PTMs

During MS/MS acquisition, peptide precursor ions within a narrow *m*/*z* range are isolated and subjected to collision induced dissociation (CID) to produce fragment ions [21,23]. As the name implies, peptide precursor ions undergo multiple collisions with inert gas in a discrete chamber (i.e., collision cell) of the mass spectrometer, leading to vibrational excitation and peptide fragmentation. In the case of unmodified peptides, CID tends to drive cleavage of backbone amide bonds via gas-phase rearrangement. MS/MS is performed on large numbers of ions for a given peptide precursor, and each amide bond in a peptide is, generally, equivalently susceptible to gas-phase cleavage [23]. As a result, MS/MS spectra often encompass dissociation of most amide bonds within a peptide, thus enabling assignment of the peptide’s sequence.

Modification of peptide side chains can dramatically alter gas-phase dissociation behavior. For instance, ester-linked modifications may undergo dissociation before backbone fragmentation [21]. Here again, phosphorylation is a prime exemplar, although many other modifications are also subject to these confounding effects. While not all modifications undergo fragmentation, those that do generally fall into one of two categories: (1) Their linkage to the amino acid side chain is labile compared to the peptide backbone, such that the entire modification is lost during MS/MS before meaningful amide bond fragmentation; or (2) they themselves fragment, which can dramatically alter the nature of peptide MS/MS bond cleavage.

In the former case, highly labile modifications (e.g., O-sulfonation) [19] dissociate completely from the peptide precursor ions and therefore do not yield fragment ions that bear the modification. In contrast, other labile modifications, such as phosphorylation and synthetic modifications generated by electrophilic probes [21,24], undergo only partial dissociation, allowing modified fragment ions to be detected. In the latter case, modifications such as acylation or glycosylation may internally dissociate independently of the amide backbone to produce fragment remnants, or modification remnants of variable mass on the peptide fragment ions. These dissociation pathways result in unexpected fragment ions in the corresponding MS/MS spectra. Most sequence assignment algorithms penalize confidence scores when MS/MS spectra contain unexplained fragment ions. As a result, it is important that users associate non-canonical dissociation pathways with specific PTMs to ensure acceptable peptide-PTM identification rates.

It is important to note that modern mass spectrometers may be equipped with different technologies to perform MS/MS that can affect a modification’s fragmentation behavior. In general, ion trap instruments utilize on-resonance excitation of peptide precursors in a partial pressure of ~1 mTorr helium. In this mode, peptides undergo numerous, sequential low-energy collisions with helium whereby their internal energy incrementally increases with each collision. With this mechanism, labile modifications may be lost before cleavage of peptide amide bonds, leading to poor PTM peptide identification rates. In contrast, so-called hybrid instruments (e.g., tribrid Orbitrap or QTOF geometries) physically separate peptide isolation and collision [25,26]. Here, after isolation, peptide precursors are accelerated to higher kinetic energy and then introduced into a collision cell for ‘beam-type’ collisional excitation. The nature of collisions in these instruments imparts higher internal energy to achieve effective amide bond cleavage even when peptides harbor labile PTMs. Electron-mediated dissociation is an alternative to collision-induced methods. With this approach [23], direct electron capture (electron capture dissociation, ‘ECD’) or transfer of an electron from a separate reagent anion (electron transfer dissociation, ‘ETD’) increases peptide internal energy non-ergodically such that backbone Cα–N bonds are preferentially cleaved with labile amino acid side chain modifications remaining largely intact. However, compared to collision-based methods, ECD/ETD is biased toward detecting large, multiply charged precursors and is characterized by somewhat slower cycle times, which combine to limit peptide identification depth in analysis of complex proteomes (see excellent reviews [23,27]). Consequently, electron-mediated dissociation remains valuable for stabilizing large or fragile modifications (e.g., glycans) and sequence analysis of large polypeptides, while beam-type CID is more widely used in bottom-up PTM analysis.

**Figure 3:**
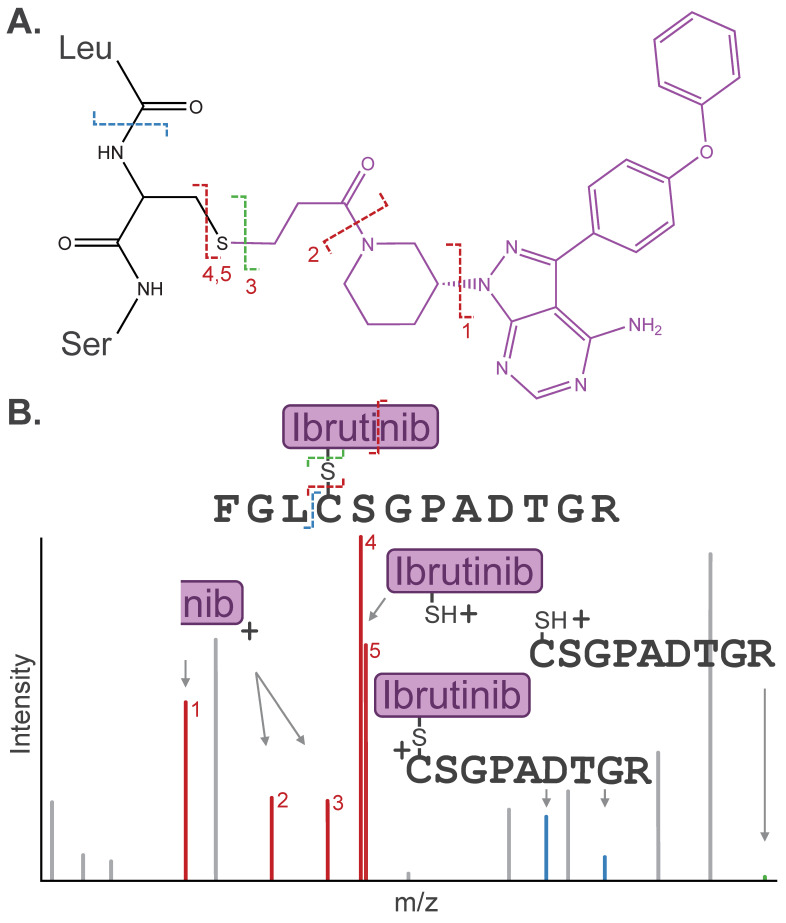
(**A**) Dissociation pathways observed for cysteine-containing peptide modified by the covalent drug Ibrutinib. (**B**) Stylized MS/MS spectrum representative of the inhibitor-related fragment ions, including diagnostic ions (red), fragment remnants (blue), or “unmodified” fragment ions (green), that are often detected in a similar intensity range as canonical peptide fragment ions (gray). Diagnostic ions for Ibrutinib or Ibrutinib internal fragmentation products (“nib“) are labeled (1–5) by their respective Ibrutinib dissociation pathways depicted in (**A**).

Post-acquisition, accounting for PTM-mediated alterations in fragmentation behavior in database search is essential for accurate identification of modified species. Identifying the dissociation pathway is a key first step. As described above, the modification may dissociate cleanly from the peptide during MS/MS, leaving the “unmodified” fragment as the most abundant detected ion in the resulting MS/MS spectrum. Alternatively, the modification may fragment internally or co-fragment with the peptide side chain (e.g., water loss on pSer/pThr) [21,28]. The resulting fragment remnants must be assigned to confidently identify the peptide sequence and site of modification (Figure 3).

Internal modification dissociation pathways have been leveraged to distinguish isomeric modifications that are otherwise indistinguishable by MS. For example, malonic acids exhibit a characteristic CO_2_ loss during MS/MS, which generates signature fragment remnants for malonylation and helps to differentiate methylmalonylation from succinylation, an isomeric acylation event [29,30]. Distinct fragment remnants have been applied to distinguish other isomeric PTMs, such as asymmetric vs. symmetric arginine dimethylation [31].

Equally critical to PTM identification is understanding the propensity of a modification to dissociate under specific experimental and acquisition conditions, as this directly informs the selection of appropriate database search tools and parameters. For instance, the use of isobaric labeling reagents has been reported to increase phosphorylation dissociation at phospho-tyrosine sites [32]. Similarly, photoaffinity labeling (PAL) warheads have been shown to preferentially react with acidic residues to form labile ester linkages, which may completely dissociate and confound sequence assignments with conventional modification searches [33]. Predicting the extent of dissociation given these considerations can be used to tailor search parameters; however, more flexible search tools like MSFragger’s “Labile” module may be used to identify modification events even when the extent of dissociation is uncertain [28].

Importantly, once characterized, the fragmentation behavior of the PTM can be leveraged to improve sensitivity for the detection of modified peptides. Modifications can dissociate from the peptide as a neutral molecule (i.e., neutral loss) that is not detected by the mass spectrometer during MS/MS. However, if the dissociated modification retains charge, the resulting diagnostic ion can be used as evidence of a modified precursor. Diagnostic ions of some modifications (e.g., phosphorylation) are established, while common dissociation pathways can be applied to predict diagnostic ions for other modifications, such as synthetic PTMs. For example, our lab has identified diagnostic thiolate ions common to cysteine-directed electrophilic probes. During MS/MS, the thioether linkage cleaves such that the intact probe dissociates as a charged fragment ion from the peptide, carrying with it the cysteine side-chain sulfur [24]. Similarly, immonium ions are caused by internal fragmentation of a single amino acid side chain from the peptide backbone under many activation techniques and have been used as diagnostic ions to improve the detection of modification events such as phosphotyrosine, acetyllysine, and lactyllysine [34,35].

Diagnostic ions can be specified in search tools such as Byonic [36] and MSFragger [37,38] to improve the detection of modified peptides. Furthermore, precursor ion scanning enables gas-phase enrichment of modified peptide species during acquisition by focusing MS/MS scans on peptides that appear to generate diagnostic ions. This approach has been utilized to increase coverage of high-confidence identifications for several PTMs, including phosphorylation, asymmetric dimethylation, and probe labeling events [24,35,39,40,41].

Efforts to computationally automate the characterization of dissociation pathways have accelerated the identification of diagnostic ions. MSFragger’s “Diagnostic Ion Mining” module analyzes MS/MS spectra for peptide sequences carrying the same modification to identify recurrent, abundant diagnostic ions as well as fragment remnants that may be utilized to improve confidence scores [38,42]. This approach has been utilized to identify biotin-triazole fragmentation products in clickable biotin reagents and confirm loss of PAL moieties linked to carboxyl side chains [43,44].

## 4. Challenges in Localization of PTM Sites

Confirming the site of modification is crucial before proceeding with mechanistic or clinical studies. For endogenous PTMs, understanding the confidence for localizing a modification can be used to triage reliable biomarker sites. For synthetic modifications, accurate localization of the modified residue enables assignment of small molecule binding sites and can accelerate hit-to-lead prioritization for the drug development pipeline. For this reason, it is critical to understand the challenges and limitations of localizing peptide modifications by MS before nominating a specific site for focused, orthogonal validation studies.

**Figure 4:**
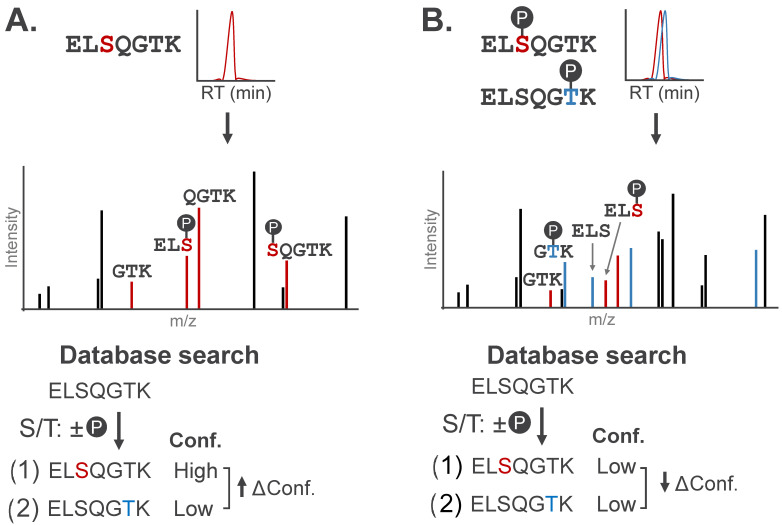
Stylized MS/MS spectra representative of singly- and multiply-phosphorylated peptides. Site-determining fragment ions can localize modification(s) to specific amino acids. (**A**) MS/MS spectrum for a phosphopeptide with site-determining fragment ions (red). Candidate peptide sequences are assigned confidence scores (Conf.) based on the number of site-determining ions, with localization confidence (Δ Conf.) reported as the difference in scores between the top two candidate sequences. (**B**) Chimeric MS/MS contains contradictory site-determining ions that diminish localization confidence (↓ Δ Conf.) of conventional search algorithms.

A primary consideration for MS-based PTM localization is the number of amino acids within a given peptide sequence that could bear the modification. If only one residue on a peptide sequence can bear a modification, assigning the site of modification is inherently unambiguous [45]. When multiple residues could theoretically bear a modification, localization relies entirely on the detection of site-determining ions, or fragment ions that carry the modification and can distinguish the modified amino acid from candidate sites on other regions of the peptide (Figure 4A). These ions usually span amide bond fragmentation events at, or immediately adjacent to, the modification site.

The extent of spectral evidence required to generate sufficient site-determining ions for confident localization depends on the modification’s labeling profile, or the scope of amino acid residues that can be modified. Some modifications, like acylation (K) and cysteine-directed electrophilic moieties (C), are largely constrained to a single residue, while others, such as phosphorylation, can occur on several residues (S/T/Y). Modifications with broad labeling profiles complicate localization efforts, as there are fewer site-determining ions capable of mapping the modification relative to other sites on the peptide. This leaves the site of some modifications ambiguous. Accounting for broad labeling profiles can be a key challenge for the identification of certain synthetic modifications, such as PAL probe adducts that can promiscuously label any amino acid.

**Figure 5:**
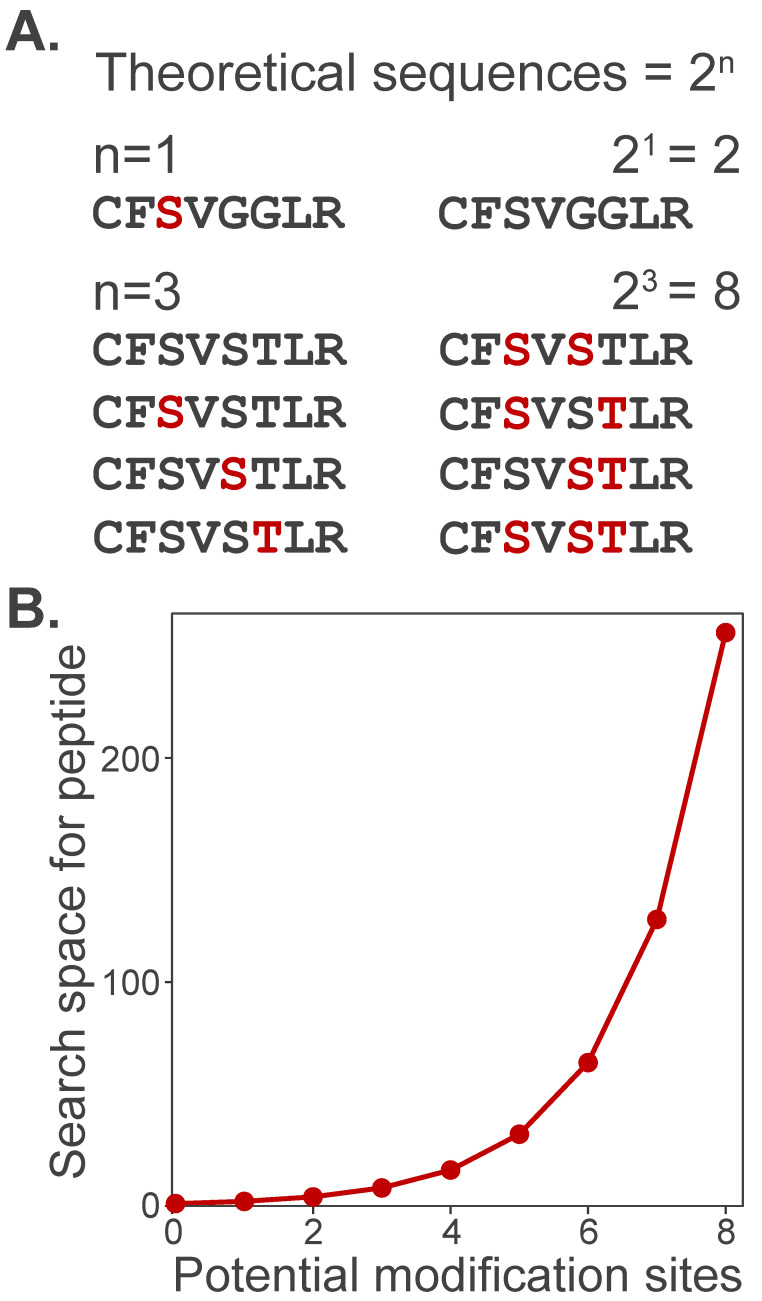
Proteome search space expansion increases with the number of potential modifications. (**A**) A peptide with only one potential modification site (e.g., phosphorylation on S/T/Y) can take only two possible forms: unmodified (black) or modified (red). (**B**) The number of theoretical peptide sequences considered by a search algorithm increases exponentially, $2^{n}$, where n is the number of potentially modified sites.

Broad labeling profiles also computationally challenge identification by expanding the search space, or the total number of candidate peptide sequences that can be matched to experimental spectra. To illustrate this, consider how increasing the number of potential modification sites expands the search space for a single peptide. A peptide containing a single modifiable site has only two theoretical sequences: modified or unmodified (Figure 5A). However, as the number of modifiable sites grows, the number of theoretical sequences increases by 2^n^. Search space grows exponentially as multiple potentially modified amino acids are specified in search algorithms (Figure 5B). This dramatic increase in search space slows search times and expands the number of theoretical sequences as potential matches, thereby increasing the number of false-positive identifications.

Identification of site-determining ions can be further complicated by labile modifications, where the modification may be partially or completely lost during MS/MS. For example, complete modification loss during CID will produce “unmodified” fragment ions, preventing MS/MS-based localization. If the modification is known to fragment internally or co-fragment with the peptide side chain, the mass of the expected fragment remnants can be used as site-determining ions to increase the number and confidence of localized modifications [28,46]. However, mass overlap between modification-induced losses and dissociation of unmodified residues can complicate analysis (e.g., phosphoric acid loss vs. water loss on unmodified S/T). The inclusion of fragment remnants to augment localization depends on the algorithm and user specifications, but should be considered when choosing a database search package and interpreting the search results.

Successful localization ultimately requires the user to understand (1) the labeling profile of the modification and (2) the expected mass shift of fragment ions that carry the modification. Algorithms perform PTM localization by comparing the intensity and number of site-determining ions that match to each potential modification site within a peptide sequence. The output of these algorithms provides the most likely site of modification along with a confidence score based on the quality of spectral evidence supporting localization at this site. Detailed discussion of PTM localization algorithms, including comparison of specific localization scoring tools and decision-making criteria for practitioners, is described in other excellent reviews [21,45,47]. As outlined, these confidence scores typically report either (1) the probability that spectral noise is falsely assigned as a site-determining ion (e.g., PTM-score of Andromeda), or (2) the reduction (delta) in confidence between the top-scoring and next-best site (e.g., Mascot MD-score, Byonic DeltaM).

Due to the limited number of site-determining ions in each spectrum, evidence for localizing a modification is often less robust than identification of the primary amino acid sequence. Poor localization confidence scores for modified peptides may arise due to low signal level (i.e., low peptide abundance) or limited fragmentation, resulting in fewer site-determining ions. In large-scale studies, these peptides would likely be de-prioritized for further validation efforts in favor of those with high-confidence assignments.

Confidence scores that capture this ambiguity in localization allow users to adjust their prioritization strategy based on the goals of the study. For example, the open-source tool PTMProphet reports normalized probabilities of a modification at each potential site as opposed to reporting only the top-scoring site [48]. Accounting for this ambiguity can, in fact, be necessary for modifications with broad labeling profiles, such as PAL probes. As PAL probes are capable of modifying any amino acid, it is unlikely that spectral evidence will resolve a PAL modification to a single residue. To adapt for this, the MSFragger: PAL workflow reports relative confidence scoring across PAL-modified sequences, highlighting regions (<3 residues) of the peptide that are likely to bear the modification given the spectral evidence. While such results are less precise, they can nonetheless be used to pinpoint binding sites of small molecule PAL probes [44].

Peptides carrying multiple modifications further complicate MS acquisition by increasing the likelihood of co-eluting positional isomers, or peptides modified on distinct sites with a shared amino acid sequence. These peptides can co-elute due to shared physicochemical properties and be co-isolated for simultaneous fragmentation; the resulting chimeric spectra will contain fragments from both peptides. Chimeric spectra impede PTM localization as evidence of both peptides exists in the MS/MS spectra, producing contradictory site-determining ions (Figure 4B).

LC platforms and MS acquisition parameters can be leveraged to improve the identification of positional isomers. For instance, previous work from our lab demonstrated the capacity of alternative chromatography modes, such as ZIP-HILIC or ERLIC, to resolve multiply phosphorylated positional isomers of RNA Polymerase II [49]. Similarly, positional isomers that may have identical chromatographic properties could be orthogonally separated by ion mobility spectrometry (IMS) techniques, such as trapped (TIMS) [50] or high-field asymmetric-waveform (FAIMS) [51]. Dynamic exclusion parameters in the MS acquisition scheme, defined as the time after a precursor is selected for MS/MS before it can be selected again, can also be optimized to resample overlapping positional isomers as they co-elute. This approach has been utilized to increase the detection of PAL-modified peptides [52].

Chimeric spectra represent a challenging case for data processing pipelines as they reduce the confidence scores for localization (Figure 4B). This issue is particularly relevant for delta-based localization confidence scores, where there is real spectral evidence supporting assignment at multiple modification sites. PAL-modified peptides are a prime example of this challenge for synthetic modifications. PAL probes stochastically modify adjacent residues in the same binding pocket, resulting in several co-eluting positional isomers during MS analysis. For this reason, it is common to relax the false discovery rate threshold in database searches for PAL modifications (1% vs. 5%) to capture proximal modification events that may have lower confidence scores [33,52,53]. However, some approaches leverage chimeric spectral features to promote the identification of these peptides. The DIZCO (diazirine probe-labeled peptide discoverer) pipeline uses chimeric spectra as a diagnostic feature of PAL-modifications, nominating sequence matches with small delta-based confidence scores as potential PAL-modified species [54]. More broadly, the DDA+ workflow in MSFragger matches multiple peptide sequences to chimeric MS/MS spectra, using precursor co-elution as additional evidence to support the assignment. This search strategy has been found to increase peptide identifications while maintaining control of the false discovery rate [55].

Both experienced practitioners and researchers of the broader scientific community need to consider localization confidence scores when nominating PTM sites from MS data. Researchers should consult reported confidence scores in published results or database search outputs to prioritize high-confidence modification events. A low confidence score does not exclude the possibility of the site being correctly assigned but rather highlights limitations in one or more of the tools comprising the data processing pipeline. This could be caused by a lack of evidence due to poor-quality MS/MS or contradictory evidence due to chimeric MS/MS spectra. Depending on the goals of the study, such cases may warrant careful, manual inspection of the data before validation or further investigation.

## 5. Analytical Validation of PTM Sites

For a modification event of interest, it is critical to confirm the target peptide’s sequence, the chemical identity of its modification, and the site of modification. Analytical validation approaches corroborate this information for ‘hits’ from MS-based PTM discovery experiments that may have been assigned with limited spectral evidence due to either low target abundance or the stochastic nature of MS acquisition. More broadly, these approaches can elucidate the signature effects of a specific modification type on a peptide’s physicochemical properties, enabling large-scale “-omics” profiling efforts (phosphorylation, ubiquitylation, etc.). For this reason, analytic validation is particularly necessary for novel or poorly characterized modifications, such as acylation and structurally diverse synthetic modifications, that lack signature MS behaviors to reference. Developing efficient strategies to rigorously characterize and validate novel modifications remains an active area of innovation in both experimental design and data analysis.

Manual assignment of MS/MS spectra should serve as the initial step in analytic validation of hits identified from PTM discovery experiments. With this approach, practitioners can utilize contextual judgement of modification-specific physicochemical properties, such as fragmentation lability, to distinguish true modifications from isomeric PTMs or analytical artifacts. This helps exclude database-driven misassignments arising from poor spectral quality or noise. Manual sequence assignment can be facilitated by both database search outputs and publicly available, manual sequencing tools [56]. Ultimately, manual inspection is minimally labor-intensive, requiring no additional sample preparation or data acquisition, and can be applied retrospectively to published PTM datasets with deposited raw MS data.

Experimental validation of modified peptides often involves strategies to increase target peptide abundance to improve spectral quality and subsequent confidence of assignment. For example, this can be achieved by immunoprecipitating a target protein to enrich for a specific modification site. For covalent small molecule probes, target engagement can be validated using purified protein for in vitro binding assays, where the compound modification event is abundant even if labeling efficiency is poor. Modification events of interest can be detected with greater sensitivity using targeted acquisition methods such as parallel reaction monitoring (PRM) [57]. PRM selectively isolates specified precursor peptides based on their expected mass-to-charge (*m*/*z*) and chromatographic retention for improved MS/MS sampling of a limited set of validation targets. This improved sampling usually translates into more robust sequence assignment and quantification of peptide targets across experimental conditions.

**Figure 6:**
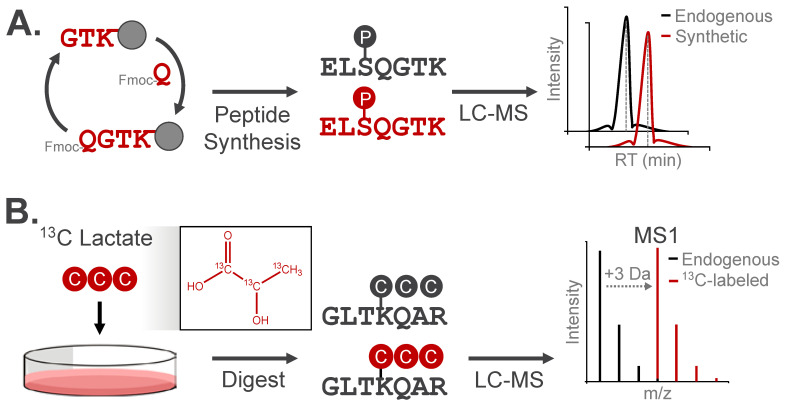
Analytical validation approaches for modified peptides. (**A**) Synthetic peptide analogs can be used to confirm LC retention, *m*/*z*, and MS/MS fragmentation behavior of experimentally observed modified peptides. (**B**) Metabolic incorporation of isotopically-enriched small molecules can be used to validate endogenous PTMs. Here, the peptide mass will increase by the number of heavy isotopes, while the LC retention and MS/MS fragmentation pattern will match the experimentally observed modified peptide.

A definitive approach for confirming the chemical identity and localization of a modification is to compare the chromatographic elution profile and fragmentation pattern of the candidate modified peptide to a synthetic analog (Figure 6A). As validation, these analogs should have identical physicochemical properties as the observed modification, with the option to spike the analog into samples for direct comparison with the endogenous modified species [58]. Synthetic peptides have been used to confirm the retention of multiply-modified phosphorylated positional isomers under different chromatographic separation approaches [49]. This strategy is particularly useful for distinguishing acylation modifications from alternative isomeric species, which cannot be resolved by mass analysis alone. For instance, HPLC-MS/MS co-elution experiments have confirmed the identity of lysine succinylation and 2-hydroxyisobutyrylation among isomeric candidate modifications [30,59].

Isotopic labeling is a widely used strategy to validate the origin of a modification by increasing the modification’s mass while preserving its chemical structure/identity. The resulting isotopic distribution produces a distinct spectral signature, which can be detected through the use of purpose-built computational tools or with manual assignment (Figure 6B). For example, tracing ^13^C-labeled lactate into +3 Da lactylated peptides confirmed the chemical identity of this modification in cells [60]. This approach has validated several acylation events, demonstrating installation of metabolite-derived modifications on lysine residues in vivo [30,60,61].

The emergence of covalent fragment screening by mass spectrometry represents a special case for dealing with synthetic modifications. These chemoproteomic approaches involve screening a library of typically >100 cysteine-directed small molecules to identify the subset of covalent compounds that bind with sufficient stoichiometry (i.e., >25%) at a given thiol side chain. Full characterization of the physicochemical properties forall compound-derived synthetic modifications in a library would be cost- and time-prohibitive, particularly since only a very small set of hits are identified in a screening campaign. As a result, these screens typically rely on “reporter” probes used in a competitive binding assay format to indirectly read out the binding behavior of all library compounds. In this way, any added effort to validate direct binding through the identification of a compound-modified peptide is focused on the limited subset of hits from the primary screen [62].

## 6. Modification-Agnostic PTM Analysis

While many of the discussed innovations have been deployed to characterize a single PTM, other “modification agnostic” approaches have been specifically developed to be compatible with a diverse set of modification chemical identities. Some database search algorithms are designed to identify potential modifications in a less restricted manner. For example, error-tolerant searches enable iterative data searching of many potential, well-defined modifications and are useful where researchers aim to discover unanticipated modifications from an MS experiment. However, these searches are limited to previously characterized modifications. Open search, or mass-tolerant search algorithms, identify undefined modifications by widening the allowed mass discrepancy (ΔM) between an experimentally observed precursor and a theoretical precursor sequence (>100 da) [37,63]. This relaxed precursor filtering retains the correct theoretical peptide sequence in a database search despite a large ΔM to the modified species, enabling comparison of MS/MS spectra comprising a range of modifications. As modified peptides often produce a subset of fragment ions that are identical to their unmodified counterparts, these search algorithms can assign peptide sequences from the MS/MS of a modified peptide without specification of the modification’s mass [63]. Reported ΔM of an assignment corresponds to the putative modification’s mass and can be cross-referenced with public modification databases such as UniMod [64] to annotate modification identity from peptide-spectrum matches [42,65]. More recent open search algorithms facilitate site localization of putative modifications by utilizing a precursor subtraction correction for assignment of modified fragments [66]. Open search pipelines have enabled the discovery of novel PTMs, such as lysine itaconylation [67].

Because open searches identify peptide modifications in an untargeted manner, their results require more stringent and intensive interpretation than approaches to profile a specific modification (e.g., phosphorylation). Reported ΔM are susceptible to mass discrepancies introduced during MS acquisition, including precursor mass error [37] or isotope misassignment [68]. Therefore, post-processing algorithms, together with chemical knowledge, are necessary to resolve a discrete modification from the aggregate ΔM distributions produced by open searches [42,68]. Interpretation becomes increasingly challenging for peptides bearing multiple modifications, for which few tools exist to deconvolute separate modifications from a single reported ΔM. Without careful interpretation, ΔM values arising from mass artifacts, amino acid insertions or substitutions, or multiply modified peptides may be misattributed to PTMs [20,69].

While modification-agnostic search tools are valuable for initial identification of modified peptides, their depth of coverage is inherently lower than PTM-specific profiling approaches. Experimentally, modifications may go undetected without enrichment strategies incorporated into PTM-specific “-omics” workflows. Computationally, modification-agnostic search tools trade identification sensitivity for flexibility; similar to expanding the number of potential modification sites, open search algorithms broaden the search space for observed spectra. This ultimately increases the relative proportion of false peptide-spectrum matches in a given search result. Indeed, direct comparisons between open searches and phosphorylation-specific searches of samples from complex proteomes have demonstrated a >2-fold improvement in sensitivity when specifying phosphorylation as a discrete modification [63].

A further limitation of modification-agnostic tools is that they only report the mass of a potential modification without other identifiers to infer its chemical identity. In contrast, once a modification is well-characterized, its physicochemical behavior, such as the propensity to produce diagnostic ions during MS/MS, can be incorporated into the parameters of a modification-specific database search to strengthen confidence in identification. For this reason, any putative modifications identified through modification-agnostic searches must be analytically validated before downstream biochemical or functional investigation.

## 7. Conclusions

Despite the substantial progress outlined in this review, significant gaps remain in our understanding of protein modifications that highlight the need for further advances in MS-based PTM analysis. An underlying goal in the study of endogenous modifications is understanding PTM crosstalk, or the interplay of distinct PTM types in physiologic and pathologic cell processes. However, it can be challenging to profile this crosstalk with conventional PTM workflows that are tailored for individual modifications. While search tools and experimental workflows for simultaneous, multi-PTM analyses are emerging, achieving comparable sensitivity of identification to these tailored approaches remains a key hurdle. Meanwhile, systematic reanalysis of published MS datasets has the potential to identify co-regulatory PTM networks, particularly as standardized reporting of experimental design and acquisition parameters becomes more widespread among MS practitioners. For synthetic modifications, a major bottleneck lies in characterizing signature MS behaviors across chemically diverse probe-derived modifications, a process that remains labor-intensive and poorly translatable between modifications. Advances in chemoproteomic reagents, sample preparation, and automated annotation of signature MS behaviors have the potential to improve throughput and reproducibility of profiling these synthetic modifications within the proteome. Ultimately, MS-based PTM analysis remains a powerful strategy for profiling both endogenous and synthetic protein modifications, enabling the discovery of disease biomarkers and ligands for disease targets. While these MS-based approaches are versatile, each type of modification introduces distinct physicochemical properties that can confound accurate identification if not considered as part of the overall experimental study design. Knowledge of modification-specific behavior can enhance PTM identifications at multiple stages of a PTM profiling workflow, including LC separation, MS acquisition, and database searching. For published PTM datasets, confidence metrics help the broader scientific community assess the validity of reported modification events before follow-up studies. By understanding how MS approaches nominate PTMs and how individual modifications influence this process, researchers can successfully integrate MS-based PTM analysis into investigations of disease targets.

## Data Availability

No new data were created or analyzed in this study.
